# Elevated expression of IFN-inducible CXCR3 ligands predicts poor prognosis in patients with non-metastatic clear-cell renal cell carcinoma

**DOI:** 10.18632/oncotarget.7468

**Published:** 2016-02-17

**Authors:** Weisi Liu, Yidong Liu, Qiang Fu, Lin Zhou, Yuan Chang, Le Xu, Weijuan Zhang, Jiejie Xu

**Affiliations:** ^1^ Department of Biochemistry and Molecular Biology, School of Basic Medical Sciences, Fudan University, Shanghai, China; ^2^ Department of Urology, Zhongshan Hospital, Fudan University, Shanghai, China; ^3^ Department of Urology, Ruijin Hospital, School of Medicine, Shanghai Jiaotong University, Shanghai, China; ^4^ Department of Immunology, School of Basic Medical Sciences, Fudan University, Shanghai, China

**Keywords:** clear-cell renal carcinoma, CXCL9, CXCL10, CXCL11, prognostic factor

## Abstract

IFN-inducible CXCR3 ligands (ICL), namely CXCL9, CXCL10 and CXCL11, exhibit pleiotropic roles in orchestrating immunity and angiogenesis. However, the prognosis value of them in renal cell carcinoma (RCC) was still obscure. Thus, we retrospectively used immunohistochemistry approach to evaluate the impact of these ligands on recurrence and survival of non-metastatic clear cell RCC (ccRCC) patients after nephrectomy. We systemically built a prespecified ICL score based on these ligands, and found specimens with high ICL score were prone to possess high Fuhrman grade, necrosis, and high-risk level of SSIGN. Moreover, ICL score stratified patients into different risk subgroups, and remained an independent adverse prognosticator for overall survival (OS) and recurrence-free survival (RFS). Meanwhile, in TCGA database, the increasing ICL mRNA predicted poor survival and early recurrence. Furthermore, after adding ICL score into SSIGN, the C-index for OS and RFS increased from 0.705 to 0.746 and 0.712 to 0.765, respectively. In conclusion, the ICL score based on expression of CXCL9, CXCL10 and CXCL11 stratified non-metastatic ccRCC patients into different risk subgroups of recurrence and death, which might benefit preoperative risk stratification and guide immune therapy in the future.

## INTRODUCTION

Renal cell carcinoma (RCC), unlike most solid tumors, is considered an immunogenic tumor and remains one of few solid tumors that consistently respond to the currently available immunotherapies [1]. Despite the strongly infiltration of different types of immune cells, immunologic dysfunction finally promote RCC tumor growth and evasion, and contribute poor survival of patients [2-4]. Thus, recognition of immunologic dysfunction in RCC, especially clear cell renal cell carcinoma (ccRCC), the most common histological subtype of RCC, has rendered a privileged area for the development of prognostic and predictive system for stratification of patients and clinical application of precise immunotherapy.

CXC chemokine are fundamental molecules to engage different leukocyte subsets to local inflammatory sites [5]. Among them, interferon (IFN) inducible CXC chemokine, CXCL9 (Mig), CXCL10 (IP-10) and CXCL11 (I-TAC), are multifunctional chemokine orchestrating immunity and angiogenesis via shared G-protein coupled receptor CXCR3, thus, these ligands might play a crucial role in cancer [6, 7]. Circulating levels of CXCL9 was reproducibly associated with lung cancer risk [8]. Meanwhile, in breast cancer, CXCL10/CXCR3 axis presented with tumor infiltrating lymphocytes (TILs), tumor progression and invasion, and poor prognosis [9, 10]. Moreover, in RCC, pervious studies found the expression of CXCL9, CXCL10 and CXCL11 increased in tumor compared to normal kidney tissues, suggesting the association with TILs and favorable prognosis, and the promotion of tumor specific immunity in systemic high-dose interleukin-2 (IL-2) therapy [11-14]. However, recently study about TILs in RCC demonstrated not only effector T cells but also regulatory T cells could be recruited via CXCR3 ligands, infiltration of Treg indicating suppression of effector T cells and poor prognosis of RCC patients [15-18]. Furthermore, overexpression of these ligands could enhance RCC cells metastasis [19, 20]. Thus, IFN-inducible CXCR3 ligands might affect tumor microenvironment via a paracrine manner and play a role in tumor progression and invasion via an autocrine manner. However, the role and prognostic value of IFN-inducible CXCR3 ligands in non-metastatic ccRCC is perplexed.

Here, we used immunohistochemistry (IHC) approach to retrospectively assess the expression of CXCL9, CXCL10 and CXCL11 in non-metastatic ccRCC specimens. A prespecified score was developed based on their expression, and then correlations with clinic characteristics and outcomes and prognostic values in multivariable Cox models were analyzed.

## RESULTS

### Association CXCL9, CXCL10 and CXCL11 expression with clinical outcomes

**Figure 1:**
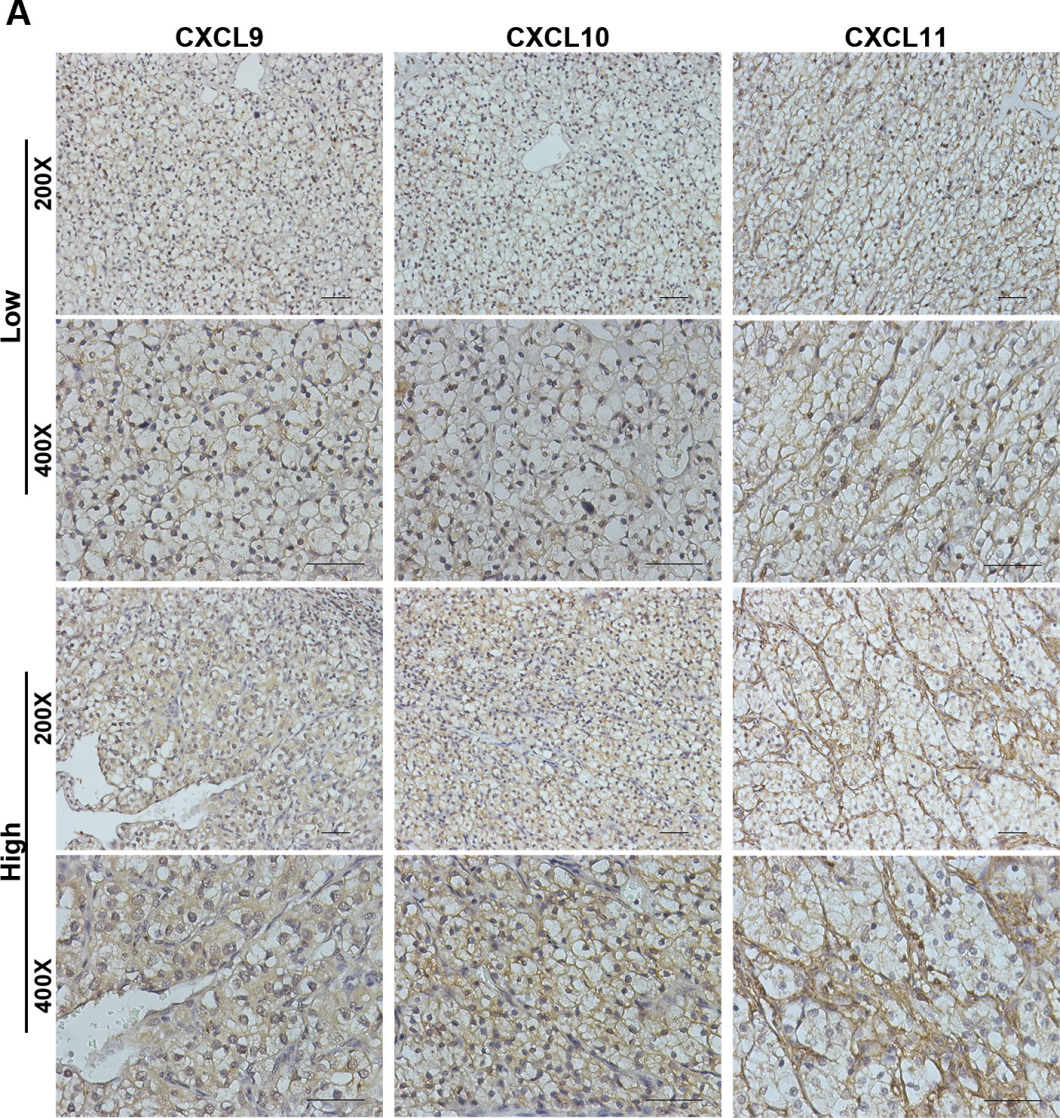
CXCL9, CXCL10 and CXCL11 immunohistochemical expression in non-metastatic ccRCC specimens (**A**) Representative CXCL9, CXCL10 and CXCL11 immunohistochemical images in non-metastatic ccRCC specimens. Scale bar: 50 μm.

CXCL9, CXCL10 and CXCL11 expression were assessed in 263 non-metastatic ccRCC specimens. We found CXCL9 and CXCL10 expressing on tumor cells and stromal cells, and CXCL11 expressing strongly on stromal cells and weakly on tumor cells (Figure 1A). According to medium value as cutoff, 122 (47.5%), 110 (42.6%) and 127 (49.4%) were grouped as CXCL9, CXCL10 and CXCL11 high expression, respectively. Furthermore, high CXCL9 expression were positive related with Fuhrman grade and SSIGN, high CXCL10 expression were positive associated with tumor size, pT stage, Fuhrman grade, necrosis and the Mayo Clinic stage, size, grade and necrosis (SSIGN) score, and high CXCL11 expression were no relevant with clinic characters in our study (Supplementary Table S1).

At last follow-up, a mean duration of overall survival (OS) was 83.3 months (media *n* = 98 months; range from 7–120 months) and recurrence-free survival (RFS) was 82.2 months (median *=* 97 months; range from 2–120 months). The patients with high expression of CXCL9, CXCL10 and CXCL11 were more likely to have poor survival and early recurrence, respectively (Supplementary Figure S1A, S1B and S1C). Thus, we built a systemic IFN-inducible CXCR3 ligands (ICL) score to evaluate prognostic value of these ligands.

### Association ICL score with clinicopathologic characteristics and clinical outcomes

**Figure 2:**
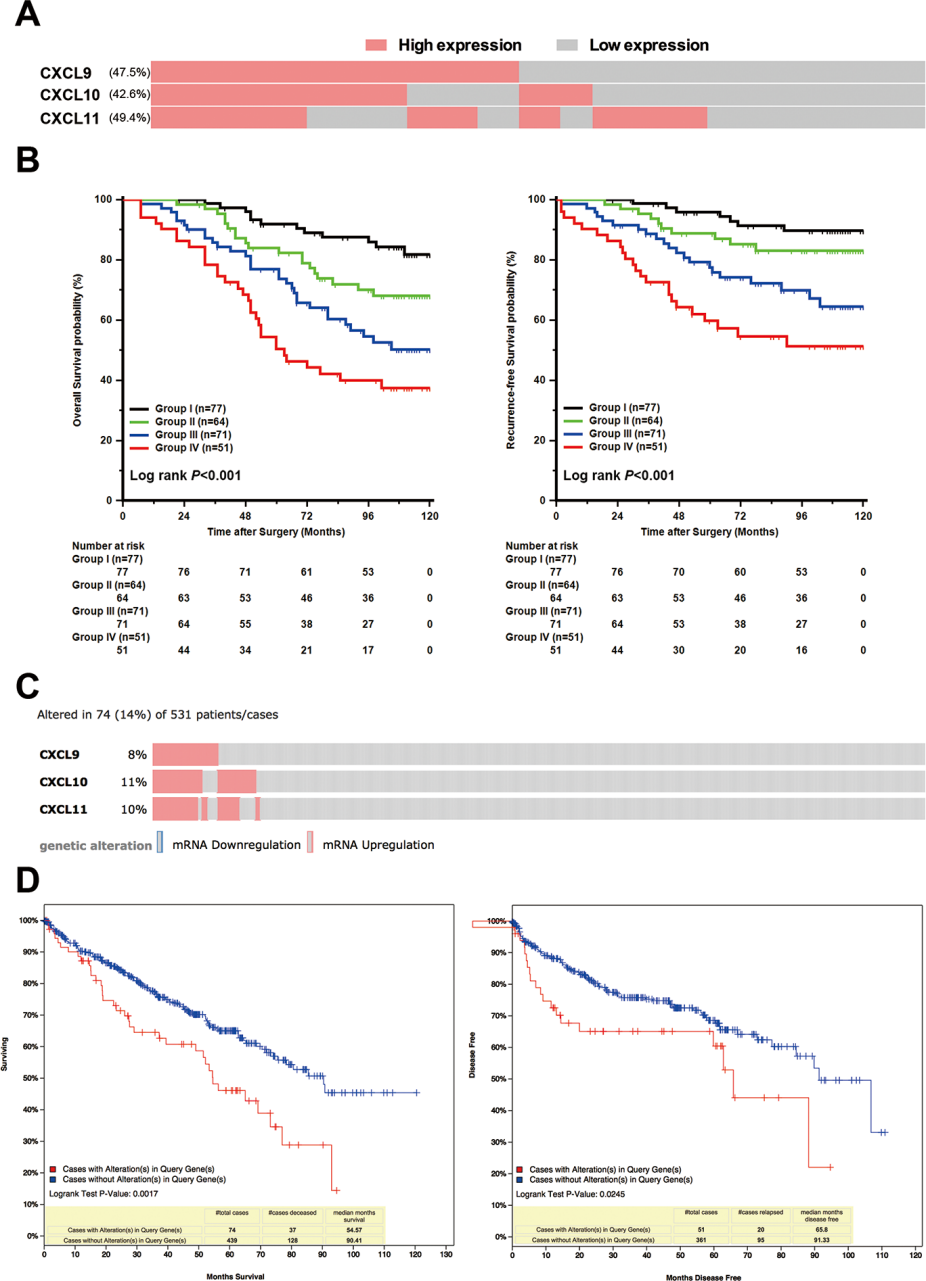
Association of prespecified IFN-inducible CXCR3 ligands (ICL) score with OS and RFS in non-metastatic ccRCC patients (**A**) Schematic diagram for the patients with different CXCL9, CXCL10 and CXCL11 expression. (**B**) Kaplan-Meier analysis of OS and RFS subgrouped by prespecified ICL score. (**C**) Schematic diagram for the patients with different CXCL9, CXCL10 and CXCL11 expression in TCGA database (z-score threshold is 0.8). (**D**) Kaplan-Meier analysis of OS and RFS dichotomized by alteration of CXCL9, CXCL10 and CXCL11.

In our prespecified ICL score, 77, 64, 71 and 51 specimens were stratified into four different subgroups, respectively (Figure 2A). The specimens with high ICL score tended to have high Fuhrman grade, necrosis, and high-risk level of SSIGN (Table 1). Furthermore, ICL score stratified patients into different risk subgroups of OS and RFS, where Group IV patients had the worst survival and earliest recurrence (Figure 2B). Meanwhile, in C-index analysis, the value of CXCL9, CXCL10 and CXCL11 was 0.631, 0.626 and 0.598 for OS and 0.644, 0.640 and 0.610 for RFS, respectively, and the value of ICL score improved to 0.681 for OS and 0.700 for RFS. To further confirm the result we observed, we used TCGA database as a validation [21, 22]. In TCGA database, totally 74 (14%) patients had high mRNA level of CXCL9, CXCL10 and CXCL11 (z-score threshold is 0.8) (Figure 2C). The patients with upregulation mRNA levels of these ligands suffered a worse survival and earlier recurrence (Figure 2D).

**Table 1:** Associations between patient characteristics and ICL Score

Characteristics	Patients	IFN-inducible CXCR3 ligands (ICL) Score				
	Total (%)	I (*n* = 77)	II (*n* = 64)	III (*n* = 71)	IV (*n* = 51)	*P*
Age (years)†						0.405
Mean	56.7	56.0	58.7	55.5	57.1	
Median	56	54	57.5	54	59	
IQR	48–67	47–66	51–67	46–65	46–68	
Gender						0.441
Male	184 (70.0)	52	50	48	34	
Female	79 (30.0)	25	14	23	17	
Tumor size (cm) †						0.122
Mean	4.6	4.2	4.4	4.8	5.4	
Median	4	3.5	4	4	5	
IQR	3–6	2.7–5.5	3–5.3	3–6	3–7	
pT stage						0.223
pT1	169 (17.5)	56	41	45	27	
pT2	33 (12.5)	10	7	10	6	
pT3	61 (23.2)	11	16	16	18	
Fuhrman grade						0.004*
1	46 (17.5)	18	16	10	2	
2	116 (44.1)	35	33	28	20	
3	67 (25.5)	18	12	20	17	
4	34 (12.9)	6	3	13	12	
Necrosis						0.027*
Absent	202 (76.8)	62	51	58	31	
Present	61 (23.2)	15	13	14	20	
ECOG-PS						0.852
0	226 (85.9)	65	57	60	44	
≥ 1	37 (14.1)	12	7	11	7	
MVI						0.602
Absent	203 (77.2)	62	46	54	41	
Present	60 (22.8)	15	18	17	10	
SSIGN						0.001*
0–3	188 (71.5)	64	52	46	26	
4–7	68 (25.8)	13	12	22	21	
≥ 8	7 (2.7)	0	0	3	4	

IQR, Interquartile range; MVI, Microvascular invasion; ECOG-PS, Eastern Cooperative Oncology Group performance status; SSIGN, the Mayo Clinic stage, size, grade, and necrosis score.

† The results were calculated by Kruskal-Wallis test.

* *P* < 0.05 is considered statistically significant.

### Multivariate analysis of prespecified ICL score with OS and RFS

**Figure 3:**
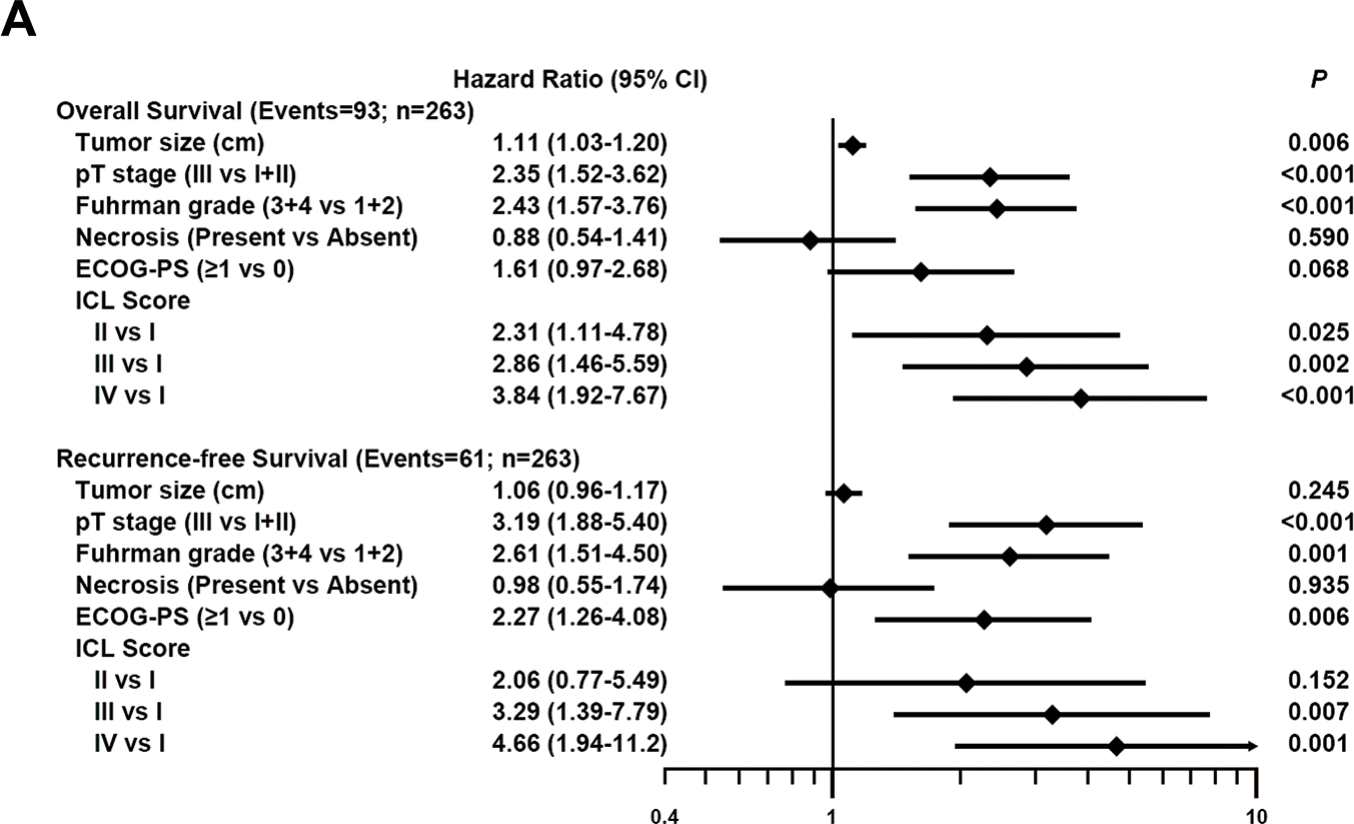
Multivariable Cox regression analysis associated of prespecified ICL score for OS and RFS (**A**) Multivariable Cox model associated ICL score with OS and RFS after adjustment for well-established variables.

To evaluate the robustness value of ICL score, multivariate Cox regression test was used to derive risk assessment correlated of OS and RFS with well-established clinicopathologic characteristics. Adjusted by these factors, prespecified ICL score remained an independent prognostic factor for OS (HR for IV vs. I = 3.84, 95% CI = 1.92–7.67, *p* < 0.001) and RFS (HR for IV vs. I = 4.66, 95% CI = 1.94–11.2, *p* < 0.001) (Figure 3A).

### Impact of prespecified ICL score on OS and RFS after adjusted by SSIGN

At last, due to the positive relationship with SSIGN, we analyzed the impact of prespecified ICL score on OS and RFS in different subgroups of SSIGN. ICL score stratified the patients with low-risk level of SSIGN in OS (Supplementary Figure S1A) and RFS (Supplementary Figure S2B). Meanwhile, ICL score remained in the Cox model for OS (HR for IV vs. I = 3.59, 95% CI = 1.79–7.20, *p* < 0.001) and RFS (HR for IV vs. I = 4.34, 95% CI = 1.80–10.5, *p =* 0.001) (Table 2). Based on the Cox analysis, the C-index of SSIGN alone was 0.705 for OS and 0.712 for RFS and improved to 0.746 for OS (*p =* 0.027) and 0.765 for RFS (*p =* 0.037) after ICL score were added (Table 2).

**Table 2:** C-index analysis based on cox model of ICL Score and SSIGN for overall survival and recurrence-free survival of non-metastasis ccRCC patients

	HR (95% CI)	*P*	C-index
**Overall Survival (Events = 93; *n* = 263)**			
SSIGN alone	1.35 (1.25–1.45)	< 0.001	0.705
SSIGN +ICL Score			0.746
SSIGN	1.27 (1.17–1.38)	< 0.001	
II vs I	2.14 (1.04–4.38)	0.040	
III vs I	2.90 (1.48–5.67)	0.002	
IV vs I	3.59 (1.79–7.20)	< 0.001	
**Recurrence-free Survival (Events = 61; *n* = 263)**			
SSIGN alone	1.35 (1.24-1.49)	< 0.001	0.712
SSIGN +ICL Score			0.765
SSIGN	1.27 (1.15–1.40)	< 0.001	
II vs I	1.86 (0.71–4.85)	0.210	
III vs I	3.23 (1.37–7.63)	0.008	
IV vs I	4.34 (1.80–10.5)	0.001	

ICL, IFN-γ-inducible CXCR3 ligands; HR, Hazard ratio; 95% CI, 95% confidence interval; SSIGN, the Mayo Clinic stage, size, grade, and necrosis score.

## DISCUSSION

Involvement of CXCR3 ligands has been observed in various angiogenesis as well as immunological disorders; presumably, these ligands might provide a useful prognostic and predictive biomarker. Here, using IHC approach, we constructed a straightforward score tool based on these ligands and found ICL score stratified non-metastatic ccRCC patients into different risk subgroups.

CXCR3 ligands/CXCR3 axis represented disparate observations, due to the CXCR3 isoform, the cell type and the microenvironment where the receptor was expressed [23]. Different CXCR3 isoform had discrepant functions; CXCR3-A promoted cell proliferation and migration, while CXCR3-B inhibited cell migration and induced apoptosis [24]. In ccRCC, the CXCR3-A to CXCR3-B ratio was higher in tumor samples than in normal kidney samples, and CXCR3 expression was associated with tumor metastasis [19]. Admittedly, the detailed mechanism of CXCR3/CXCR3 ligands axis on ccRCC development was rather obscure. However, regardless of discrepant CXCR3 situation, overexpression of IFN-inducible CXCR3 ligands predicted poor clinic outcomes of ccRCC patients in TCGA and our study, suggesting a final adverse result caused by IFN-inducible CXCR3 ligands/CXCR3 axis in patients with ccRCC.

CXCL9, CXCL10 and CXCL11 all bound CXCR3 and elicited migration of CXCR3 expressing cells *in vitro*, suggesting these ligands have redundant functions; however, different regulatory elements response to different stimuli and expression on distinct cell types indicated these ligands have different temporal and spatial patterns, accounting for the unique role of them [25, 26]. In our study, using ICL score gained a higher C-index value than using single ligand, suggesting systematic assessment of CXCL9, CXCL10 and CXCL11 status might present a more precise and comprehensive picture of functions and prognostic value of these ligands [27].

There are some limitations of our study warranting further discussion. First, given the heterogeneous nature of ccRCC and the population of our study, our conclusion might be overestimated and non-comprehensive due to these factors. Although we used TCGA database as validation, considered the different approach and cutoff methods, the further validation are warranted to confirm the stratifying function of ICL score in ccRCC patients. Second, due to small pieces of information in the original tumor using IHC approach, the readout results from patients' serum might be more convenient and reliably. Further study is necessary to determine the potential prognostic value of IFN-inducible CXCR3 ligands in serum. Third, we only focused on non-metastasis ccRCC patients. The potential prognostic value for metastatic ccRCC patients and predictive value in immune therapy need further studies to evaluate the capability of these ligands as a multifunctional biomarker for stratification and treatment for RCC patients.

## MATERIALS AND METHODS

### Patient population

A total of 263 non-metastatic ccRCC patients from, 2001 to 2004, who underwent radical or partial nephrectomy at Zhongshan Hospital, Shanghai, China, were enrolled in this study. The database included baseline clinicopathological characteristics and follow-up outcomes. The pT stage was resigned according to the American Joint Committee on Cancer 2010 TNM classification. The primary endpoint was OS with RFS as a secondary endpoint. OS and RFS were calculated from the day of surgery to the day of death and recurrence, respectively, or to the day of the last follow-up. The patients with larger necrotic and hemorrhagic area hampering the obtaining of representative area in sample or receiving preoperative neoadjuvant therapy were excluded. Ethical approval was granted by the research medical ethics committee of Fudan University.

### Tissue microarray and immunohistochemistry

Tissue microarrays were constructed as previously described [28]. Namely, formalin-fixed, paraffin-embedded tumor specimens were reviewed histologically using hematoxylin and eosin staining and two duplicate1.0-mm tissue cores from different areas were used to construct the TMA. Anti-CXCL9 CXCL10 and CXCL11 antibodies (1:100; ab9720, ab9807 and ab9955; Abcam, Cambridge, MA) were used for IHC staining. The negative controls were performed without primary antibodies. Two pathologists blinded to the clinical data evaluated the staining of each specimen. To avoid the inter-observer variability, the mean value of scores was adapted for further analysis. The staining was evaluated by semi-quantitative immunoreactivity score system, deriving from the multiplication of intensity of immunohistochemical staining (0, no staining; 1, weak; 2, moderate and 3, strong) and percentage of positive cells (1 point for each 10% increment; ranges from 1 to 10) ranges from 0 to 30. More than medium value was considered as high expression.

### IFN-inducible CXCR3 ligands (ICL) score

IFN-inducible CXCR3 ligands (ICL) score were developed based on the expression of CXCL9, CXCL10 and CXCL11 expression. The patients with no high expression of them grouped as I; with one high expression of them grouped as II; with two high expressions of them grouped as III; with all high expressions of them grouped as IV. Thus, all patients would be stratified into four prespecified risk subgroups for further analysis.

### Statistical analysis

Clinicopathologic data were compared among patients stratified by ICL score, using Kruskal-Wallis and Chi-square test as appropriate. Age and tumor size were modeled as continuous variables. Meanwhile, OS and RFS were estimated by Kaplan-Meier method and analyzed by log-rank test. In addition, ICL score was further evaluated in multivariable Cox models adjusting for well-known prognostic variables and the Mayo Clinic stage, size, grade, and necrosis (SSIGN) score, respectively. C-index analysis was preformed to compare the predictive accuracy of clinical outcomes by the parameters. Statistical analysis was preformed with SPSS statistics 22. All tests were two sided and *P* values < 0.05 were considered statistically significant.

## SUPPLEMENTARY MATERIALS FIGURES AND TABLE


