# Supplementary material for: Elevated expression of IFN-inducible CXCR3 ligands predicts poor prognosis in patients with non-metastatic clear-cell renal cell carcinoma
Source: Oncotarget. 2016 Feb 17;7(12):13976–83. doi: 10.18632/oncotarget.7468 (PMC4924692; doi:10.18632/oncotarget.7468)
Supplement: Supplementary file 1 [file oncotarget-07-13976-s001.pdf]

Elevated expression of IFN-inducible CXCR3 ligands predicts poor prognosis in patients with non-metastatic clear-cell renal cell carcinoma

Supplementary Materials

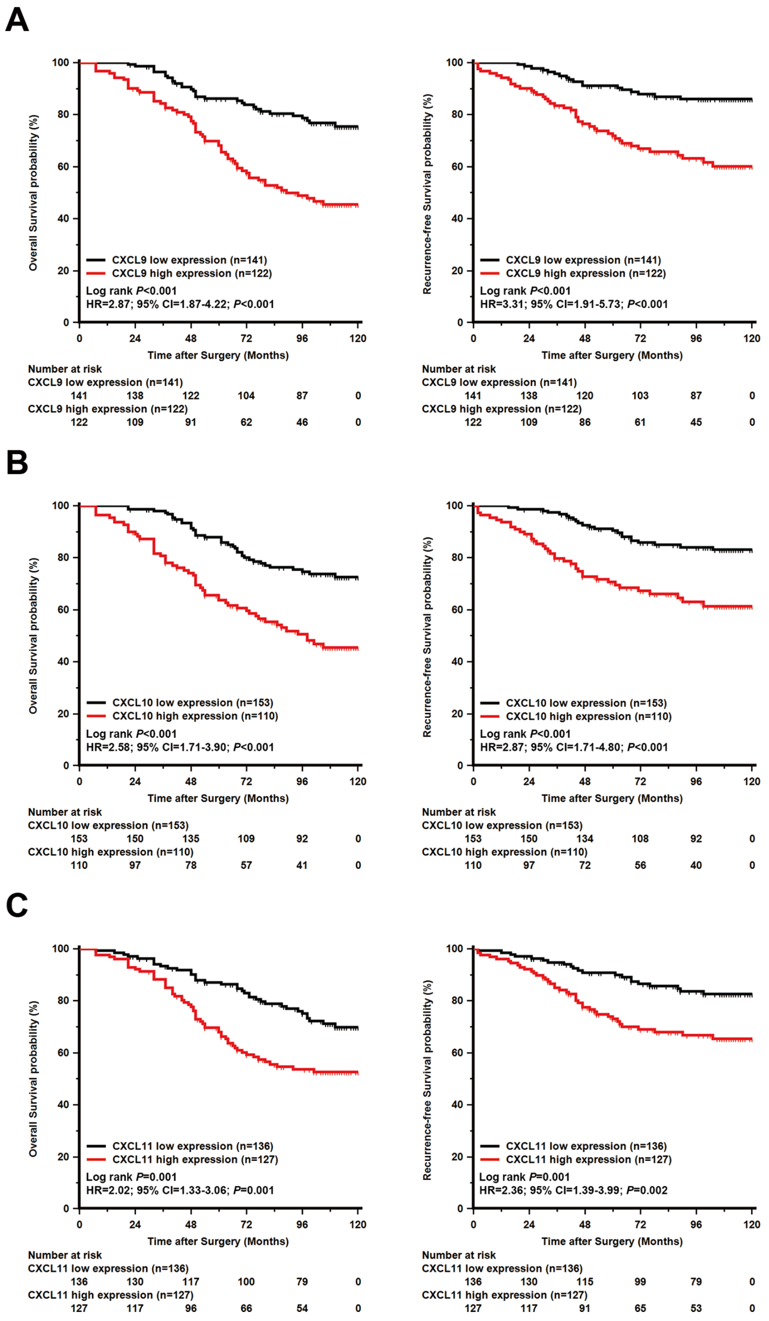

Supplementary Figure S1: Association of CXCL9, CXCL10 and CXCL11 expression with OS and RFS in non-metastatic ccRCC patients. (**A**) Kaplan-Meier analysis of OS and RFS dichotomized by CXCL9 expression. (**B**) Kaplan-Meier analysis of OS and RFS dichotomized by CXCL10 expression. (**C**) Kaplan-Meier analysis of OS and RFS dichotomized by CXCL11 expression.

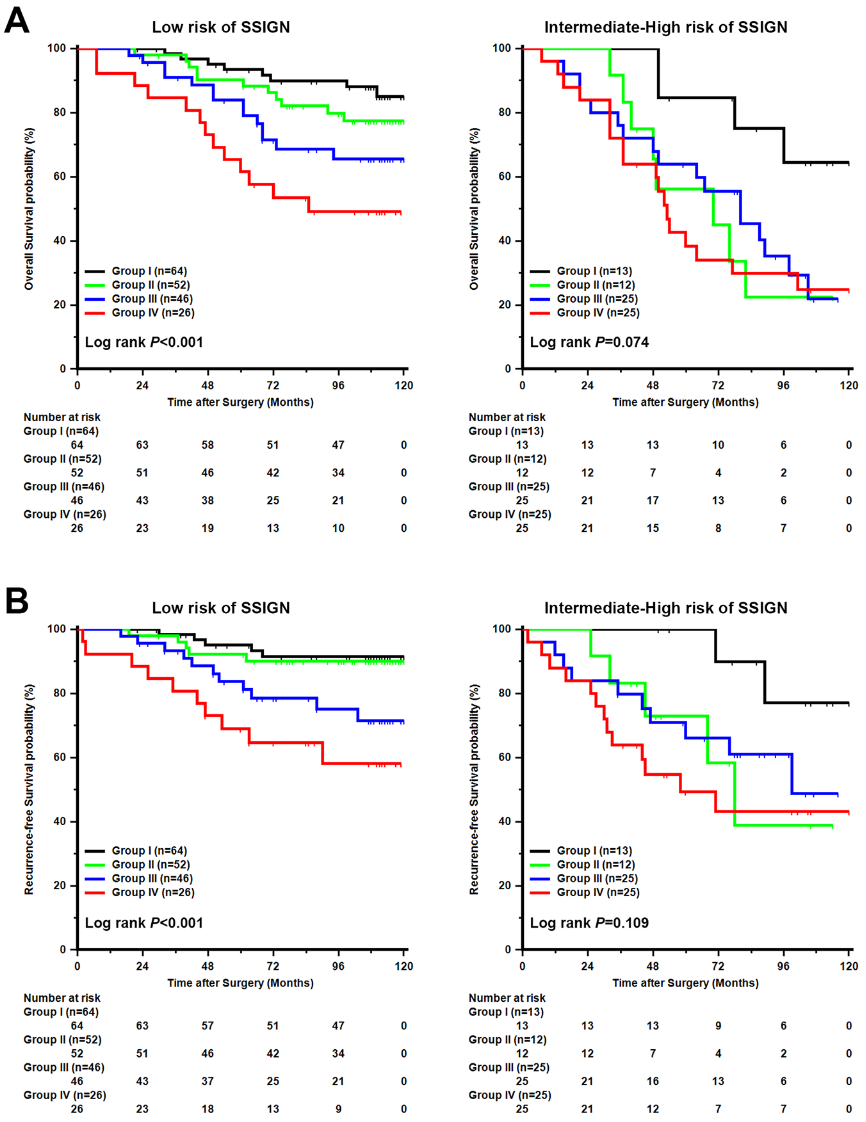

Supplementary Figure S2: Association of ICL score with OS and RFS in different subgroups of SSIGN in non-metastatic ccRCC patients. (**A**) Kaplan-Meier analysis of OS stratified by ICL score in different risk subgroups of SSIGN. (**B**) Kaplan-Meier analysis of RFS stratified by ICL score in different risk subgroups of SSIGN.

Supplementary Table S1: Associations of patient characteristics with expression of CXCL9, CXCL10 and CXCL11

	CXCL9			CXCL10			CXCL11		
	**Low** *n* = 141	**High** *n* = 122	*p*	**Low** *n* = 153	**High** *n* = 110	*p*	**Low** *n* = 136	**High** *n* = 127	*p*
Age (years)†			0.353			0.360			0.466
Mean	56.2	57.3		57.4	55.7		56.4	57.1	
Median	54	57		56	55		54	56	
IQR	49–63	48–68		50–66	45–67		47–67	50–66	
Gender			0.969			0.901			0.527
Male	99	85		107	77		98	86	
Female	42	37		46	33		38	41	
Tumor size (cm)†			0.078			0.023*			0.211
Mean	4.3	4.9		4.3	5.1		4.4	4.8	
Median	4	4		4	4.5		3.8	4.5	
IQR	2.8–5.5	3–6		3–5.5	3–6.5		3–5.5	3–6	
pT stage			0.381			0.035*			0.251
pT1	95	74		107	62		93	76	
pT2	18	15		19	14		17	16	
pT3	28	33		27	34		26	35	
Fuhrman grade			< 0.001*			< 0.001*			0.581
1	34	12		38	8		24	22	
2	67	49		67	49		65	51	
3	30	37		37	30		31	36	
4	10	24		11	23		16	18	
Necrosis			0.127			0.018*			0.760
Absent	114	88		126	76		106	96	
Present	27	34		27	34		30	31	
ECOG-PS			0.905			0.993			0.628
0	122	104		132	94		115	111	
≥ 1	19	18		21	16		21	16	
MVI			0.922			0.904			0.877
Absent	108	95		118	85		106	97	
Present	33	27		35	25		30	30	
SSIGN			< 0.001*			< 0.001*			0.096
0–3	115	73		125	63		102	86	
4–7	25	43		27	41		33	35	
≥ 8	1	6		1	6		1	6	

IQR, Interquartile range; MVI, Microvascular invasion; ECOG-PS, Eastern Cooperative Oncology Group performance status; SSIGN, the Mayo Clinic stage, size, grade, and necrosis score.

†The results were calculated by Kruskal-Wallis test.

*$P < 0.05$ is considered statistically significant.
